# Daily time-use compositions of physical behaviours and its association with evaluative and experienced wellbeing: a multilevel compositional analysis

**DOI:** 10.1186/s12966-025-01769-w

**Published:** 2025-06-11

**Authors:** Anantha Narayanan, Scott Duncan, Conal Smith, Flora Le, Lisa Mackay, Julia McPhee, Basile Chaix, Tom Stewart

**Affiliations:** 1 School of Sport and Recreation, Auckland University of Technology, Private Bag 92006, Auckland, 1142 New Zealand; 2 Kōtātā Insight, Wellington, New Zealand; 3 School of Psychological Sciences, Monash University, Melbourne, Australia; 4 Sorbonne University, Institut Pierre Louis d’Épidémiologie Et de Santé Publique, Nemesis Research Team, Paris, France

**Keywords:** Time-use epidemiology, Wellbeing, EMA, Compositional analysis

## Abstract

**Background:**

The composition of daily time-use physical behaviours—such as sedentary behaviour (SB), light physical activity (LPA), moderate-to-vigorous physical activity (MVPA), and sleep may be crucial for overall health and wellbeing. This study examined the associations between these time-use behaviours and both evaluative wellbeing (life satisfaction) and experienced wellbeing (momentary happiness, anxiousness, and tiredness). Evaluative wellbeing reflects an individual's overall life assessment, while experienced wellbeing captures real-time affective states. We investigated these associations by reallocating time among behaviours and assessing the predicted impact on wellbeing outcomes.

**Methods:**

Time-use behaviours were obtained from 211 adults who wore Axivity AX3 accelerometers on their wrists for seven days. Participants also completed a survey to assess demographics and life satisfaction, before using a custom smartphone app to report their real-time happiness, anxiousness, and tiredness levels over seven days (at three random times each day). Time-use data were processed using UK Biobank machine learning algorithms. We employed Bayesian multilevel compositional analysis to investigate how time-use behaviours, and reallocating time between behaviours, were associated with both life satisfaction and momentary affective states.

**Results:**

Increasing sedentary time (relative to other behaviours) over the week of observation was negatively associated with happiness and positively associated with anxiousness aggregated at the day level. Conversely, increasing the proportion of MVPA (relative to other behaviours) was associated with reduced anxiousness and tiredness. Substitution analysis showed that reallocating 20 min of SB to MVPA increased happiness by 0.12 units, 95% CI [0.01, 0.22] and reduced anxiousness by 0.20 units, 95% CI [-0.34, -0.07]. Additionally, reallocating 20 min of time spent in LPA to MVPA reduced tiredness by 0.16 units, 95% CI [-0.28, -0.03]. All affective states are reported on a 0–10 scale. No associations were found between time-use behaviours and life satisfaction.

**Conclusion:**

Our study shows that time-use behaviours, particularly reducing sedentary time and increasing physical activity, were more strongly linked to experienced wellbeing. Studies that focus solely on examining time-use behaviours and long-term wellbeing outcomes, such as life satisfaction (common in population studies), may overlook the dynamic interplay and immediate impacts of behaviours on wellbeing. While some associations were present, most of the tested relationships were weak or non-significant, suggesting that contextual factors like social and environmental conditions may play a greater role in shaping wellbeing. The next step is to explore sequential associations, such as behaviours occurring immediately before or after a momentary affect response is recorded.

**Supplementary Information:**

The online version contains supplementary material available at 10.1186/s12966-025-01769-w.

## Background

Regular physical activity, limited sedentary time, and adequate sleep are essential for optimal health and wellbeing across the lifespan [1]. Decades of research have established that optimising the time spent in these behaviours is fundamental for preventing chronic diseases, enhancing mental health, and promoting overall quality of life [2, 3]. Traditionally, researchers have examined the association between these behaviours and health independently, rather than collectively. Individuals can engage in a variety of behaviours throughout the day, and changes to the times spent in one behaviour inevitably affects the time that can be allocated to one or more other behaviours within a 24-h period—a paradigm often referred to as *time-use epidemiology* [4, 5]. Time-use epidemiology suggests that the composition and distribution of daily behaviours, including sedentary behaviour, light physical activity (LPA), moderate-to-vigorous physical activity (MVPA), and sleep collectively influence health outcomes beyond the effects of each behaviour in isolation. Recent studies have examined how daily time-use behaviours are associated with various health outcomes using compositional data analysis techniques [6–8]. Simultaneously, public health guidelines have also started to focus on the importance of achieving a balance in various time-use behaviours within a 24-h period [9, 10].

While much of the early research in this field has focused on the physiological and metabolic consequences of varying time-use patterns, only a few studies have explored their broader implications on wellbeing [6, 7, 11]. Subjective wellbeing (hereafter wellbeing) refers to the existence of good mental states and has both an evaluative dimension and an affective or experienced dimension [12, 13]. Evaluative wellbeing captures a person’s reflective assessment of their life as a whole, such as life satisfaction [12]. In contrast, experienced wellbeing captures real-time emotions and mental states, which fluctuate dynamically in response to immediate environmental contexts and daily behaviours, including physical activity, sleep, and social interactions [13]. Understanding how different time-use behaviours influence evaluative wellbeing is essential for developing interventions that enhance holistic health and quality of life across diverse populations. Research has shown that frequent positive emotional experiences—such as joy, calmness, or reduced stress—may accumulate over time and improve a person’s evaluative wellbeing, shaping their broader sense of life satisfaction and purpose [14, 15]. To fully grasp this relationship, it is important to investigate how daily activities affect both experienced and evaluative wellbeing. This approach allows us to uncover how changes in behaviour can impact immediate emotional states and, over time, lead to more substantial improvements in life satisfaction.

The advent of technology has significantly advanced our ability to capture experienced wellbeing data in real time, usually through Ecological Momentary Assessment (EMA). EMA involves repeated sampling of an individual’s behaviour and experiences in their natural environments through surveys, providing a rich, dynamic picture of how people feel at different times of the day. This approach complements traditional evaluative measures of subjective wellbeing, which rely on retrospective assessments that can be influenced by recall bias and may not capture the nuances of day-to-day emotional fluctuations.

Physical activity is widely recognised for its benefits to experienced wellbeing, including improvements in mood and reductions in anxiety and depression [2]. Similarly, a balanced approach to time-use behaviours can contribute to life satisfaction, reflecting broader healthy lifestyle practices [16]. Although previous research has explored these relationships separately, a key gap remains in understanding how time-use behaviours are associated with these two measures of subjective wellbeing. Therefore, this study aims to inform evidence-based strategies for promoting balanced movement behaviours that optimise both physical health and psychological wellbeing. Several studies have examined how theoretical reallocations of time among different time-use behaviours are associated with health outcomes, using compositional isotemporal substitution analysis [4, 7]. Building on this work, the primary aim of this study is to investigate the association between time-use behaviours and two distinct dimensions of subjective wellbeing, evaluative and experienced wellbeing, in a sample of urban adults.

## Methods

### Participants

This study utilised data from the *Te Hotonga Hapori (Connecting communities)* programme, which is investigating the impact of urban regeneration on community wellbeing in Auckland, New Zealand. For more details on the programme, see [17]. The data utilised in this study is specifically drawn from the baseline phase of the project, conducted between April 2023 and May 2024. A total of 481 adults from diverse ethnic backgrounds participated, with 429 consenting to wear accelerometers. Participants were required to be at least 18 years old and residents of specific neighbourhoods undergoing urban regeneration in Auckland (Wesley, Waikōwhai, Oranga, and Aorere). Recruitment involved community hui (meetings or gatherings), information evenings, and engagement at local events, facilitated in collaboration with research partners and community groups. Eligible households were also mailed information pamphlets detailing the study and participation process.

## Measurements

### Outcome measures

#### Evaluative Wellbeing (Life Satisfaction)

Before wearing the accelerometer and undergoing the smartphone survey, participants completed a wellbeing survey that included both demographic and wellbeing-related questions (based on the New Zealand government’s Living Standards Framework domains [18, 19]). This included life satisfaction, which was measured using a single-item question drawn from the New Zealand General Social Survey (NZGSS): “Thinking about your life as a whole, how satisfied are you with it?” Responses ranged from 0 (not at all satisfied) to 10 (completely satisfied). This measure has demonstrated acceptable validity [20], is widely used in population-based wellbeing research [21], and provides an overall evaluation of participants’ life satisfaction.

#### Experienced Wellbeing (Momentary Affect)

Experienced wellbeing, or momentary affect, was measured using the *Te Hotonga Hapori* smartphone application which was developed as an extension to the existing *Eco Emo tracker* platform [22]. During the initial consultation, participants were instructed to download the mobile app and to respond to the survey prompts for the next seven consecutive days. The app sent notifications randomly within three designated time windows each day: 8 am to 12 pm, 12 pm to 5 pm, and 5 pm to 9 pm, resulting in a total of 21 prompts over the week. These prompts were randomly distributed across both weekdays and weekends to ensure broad temporal coverage, although day-of-week differences in wellbeing were not specifically analysed in this study. As part of each prompt, participants were asked three questions to rate their current feelings drawing on guidelines from a recent systematic review of smartphone-based EMA studies [13] – “How happy do you feel right now?", "How anxious do you feel right now?", and "How tired do you feel right now?". Each response was rated on a scale from 0 (not at all) to 10 (extremely), with a 60-min window provided to complete each response. If they did not respond within 30 min, a reminder notification was sent. Each response was saved with a timestamp. The data collected by the app were automatically transferred to a cloud server, which the researchers accessed via a web application prior to analysis.

## Exposure measures and covariates

Time-use behaviours were objectively measured using an Axivity AX3 accelerometer worn on each participant’s non-dominant wrist for seven consecutive days, which directly aligned with the days during which EMA was conducted through the smartphone app. The accelerometers collected raw acceleration data in three axes at a sampling rate of 100 Hz. These raw data files were downloaded using Open Movement Software (OMGUI, version 1.0.0.30, Open Movement, Newcastle University, UK). Detailed specifications can be found on the Axivity website (http://axivity.com/product/ax3). Data were processed using UK Biobank machine learning algorithms [23] to derive daily estimates (in minutes) of four key time-use behaviours: Sedentary, LPA, MVPA, and Sleep. A detailed description of the Biobank accelerometer analysis methodology can be found elsewhere [24]. Days with non-wear time exceeding two hours were excluded from the analysis to ensure data accuracy and reliability of the time-use distribution indicators. Only participants who had data for at least two complete days were included in the final analysis. While studies typically consider between two and five days of accelerometer data as reliable for estimating activity patterns [25], our decision to include at least two days of accelerometer data was made to maximise the sample size while balancing data reliability. Each participant’s age, gender, ethnicity, and 2018 NZ Deprivation Index (NZDep2018) [26] – a composite measure defined at the meshblock level that assesses various dimensions of deprivation, including income, employment, and education, to evaluate the socioeconomic status of individuals and communities across New Zealand (with decile 1 representing low deprivation and decile 10 representing high deprivation) [27], were captured during the wellbeing survey and treated as covariates.

### Statistical analysis

All statistical analyses were performed using R software (version 4.3.3; The R Foundation for Statistical Computing, Vienna, Austria). Standard descriptive statistics were calculated for demographic variables. Given the compositional nature of time-use data (i.e., the total time allocated to all activities sums to a fixed total), compositional analysis techniques were employed using the *compositions* [28], *multilevelcoda* [29, 30], and *deltacomp* [31] R packages.

A four-part composition was created from the time spent in sedentary behaviour (SB), LPA, MVPA, and sleep. Firstly, missing values and parts of each composition that contained zeros were imputed using log-ratio expectation–maximisation, a method recognised for minimising bias [32]. The average daily minutes spent in each 24-h time-use behaviour were reported as both arithmetic and geometric means. The geometric mean of each behaviour was normalised to 1440 min.

Next, the compositions were expressed as isometric log-ratio (ilr) coordinates. These ilr coordinates were derived using the sequential binary partition (SBP) process [33] with the following sign matrix:

**Table:** 

	𝑥1	𝑥2	𝑥3	𝑥4
ilr1	+ 1	−1	−1	−1
ilr2	0	+ 1	−1	−1
ilr3	0	0	+ 1	−1

Here, 𝑥1, 𝑥2, 𝑥3, and 𝑥4 represent the four components of the time-use composition (SB, LPA, MVPA, sleep), and ilr1, ilr2, and ilr3 are the resulting ilr coordinates. Ilr1 represents the time spent in behaviour 𝑥1 relative to all other behaviours, while ilr2 represents the ratio of  𝑥2 relative to 𝑥3 and 𝑥4, and ilr3 represents the ratio of 𝑥3 to 𝑥4.

The ilr coordinates were calculated using the *complr* function in the *multilevelcoda* package in R. The general formula for the ilr1 coordinate is given below:

$${\text{ilr}}_{1,x}=\sqrt{\frac{D-1}{D}}\cdot \text{log}\left(\frac{x}{{\left({\prod }_{i=2}^{D}{x}_{i}\right)}^{1/\left(D-1\right)}}\right)$$

where, D = 4 in this study, indicating a four-part composition. For example, the ilr1 for SB relative to the other behaviours is:

$${\text{ilr}}_{1,{\text{SB}}}=\sqrt{\frac{3}{4}} \cdot \text{ln}\left(\frac{\text{SB}}{{\left({\text{LPA}}\cdot {\text{MVPA}}\cdot {\text{Sleep}}\right)}}\right)=\text{ln}\left(\frac{{\text{SB}}^{\sqrt{\frac{3}{4}} }}{{\left({\text{LPA}}\cdot {\text{MVPA}}\cdot {\text{Sleep}}\right)}^{\sqrt{\frac{1}{12}} }}\right)$$

More information on ilr transformations in compositional data analysis can be found elsewhere [31].

For affect analysis, daily affect (happiness, anxiousness and tiredness) was calculated by averaging participants’ EMAs per day up to three measures per participants. Daily time-use (expressed as ilr coordinates) were also decomposed into between-person and within-person levels. The between-person ilr coordinates capture the differences in the average time spent in time-use behaviours between individuals, whereas the within-person ilr coordinates represent the mean-centred deviates from an individual’s average time-use behaviours on a given day. Bayesian multilevel models were then fitted with each affect at the day level as the dependant variable (separately), while the ilr coordinates were the explanatory variables. For life satisfaction analysis, a linear model was fitted with life satisfaction as the dependent variable, while the ilr coordinates served as explanatory variables. Both analyses were adjusted for age, gender, ethnicity, and NZDep2018. This modelling process was repeated four times, each with a different permutation of the sign matrix. The sign matrix was rearranged such that each of the four behaviours (SB, LPA, MVPA, sleep) was represented as 𝑥1, and the corresponding ilr1 represented the ratio of this behaviour to the remaining three behaviours. This was performed to aid the interpretation of the ilr regression coefficients [33].

Finally, compositional isotemporal substitution was used to examine predicted differences in affect and life satisfaction associated with pairwise reallocation of time from one behaviour to another behaviour in 5-min increments (up to 20 min), using the mean time-use composition of the sample as a reference [34].

## Results

Of 429 adults who consented to wear the accelerometers, 218 were excluded due to insufficient accelerometer wear time. A total of 211 adults (71% female) were included in the final analysis, all of whom provided at least two days of valid accelerometer data and had reported both wellbeing measures. The mean age of participants was 42 years (SD = 12.3). Missing data from EMA responses were handled by excluding participants who did not complete at least one EMA response per day across the study period. The sample was ethnically diverse, comprising NZ European (40%), Māori (16%), Pacific Island (32%) Asian (23%), and other ethnicities (2.4%). Table 1 provides a detailed description of participants’ characteristics. In this selected sample, participants wore Axivity AX3 accelerometers for an average of 4.4 days (SD = 2.0). Real-time experienced wellbeing assessments were completed, with participants providing an average of approximately 8 responses (SD = 6) over the 7-day period. The mean scores reported by participants were happiness: 7.05 (SD = 2.26), anxiousness: 2.43 (SD = 2.58), tiredness: 4.29 (SD = 2.83); all scores measured on a scale from 0 to 10. The mean life satisfaction score was 7.28 (SD = 1.90) on a scale from 0 to 10.

**Table 1:** Characteristics of the study sample

Characteristics	Number (%)
Gender	
Male	60 (28.4%)
Female	151 (71.6%)
Age range	
< 25 years	23 (10.9%)
25–34 years	45 (21.3%)
35–44 years	44 (20.8%)
45–54 years	34 (16.1%)
55–64 years	40 (18.9%)
65 + years	25 (11.8%)
Ethnicity	
NZ European	85 (40.3%)
Māori	34 (16.1%)
Pacific Island	67 (31.7%)
Asian	48 (22.7%)
Other	5 (2.4%)
NZDep2018	
1 (low deprivation)	0 (0%)
2	0 (0%)
3	12 (5.7%)
4	12 (5.7%)
5	17 (8.05%)
6	20 (9.5%)
7	10 (4.7%)
8	31 (14.7%)
9	30 (14.2%)
10 (high deprivation)	79 (37.4%)

Participants were able to select multiple ethnicities, so the total percentages may exceed 100%

Table 2 summarises the arithmetic and compositional means of daily minutes spent in each time-use behaviour measured by the accelerometers. On average, participants spent 682 min in sedentary behaviour, 278 min in LPA, 8 min in MVPA, and 471 min sleeping each day.

**Table 2:** Arithmetic and compositional means of time spent in each physical behaviour

Variable	Arithmetic Mean Minutes/Day (SD)	Geometric Mean Minutes/Day (GSD)
Sedentary	659 (138)	682 (144)
Light PA	291(129)	278 (130)
MVPA	21 (13)	8 (29)
Sleep	453 (89)	471 (86)

Arithmetic means reflect the average time spent in each behaviour independently, while the geometric means account for the relative distribution of time across all behaviours

Table 3 presents the results from the multilevel compositional analysis (both unadjusted and adjusted models), which explored the daily associations between time-use behaviours and affect. The adjusted models revealed that, at the between-person level, more sedentary behaviour (relative to the remaining behaviours) was associated with lower happiness (*B* = − 1.54, 95% CI = [−3.05, −0.05], p < 0.05) and higher anxiousness (*B* = 1.89, 95% CI = 0.01–3.78, p < 0.05). Conversely, more MVPA (relative to the remaining behaviours) was associated with lower levels of anxiousness (*B* = −0.30, 95% CI = [−0.55, −0.05]) and tiredness (*B* = −0.24, 95% CI = −0.46 – −0.02) at the between-person level. However, no associations were found between daily time-use and affect at the within-person level. Further, no associations were found between time-use behaviours and life satisfaction (see Table 4).

**Table 3:** Relationship between time-use compositions (expressed as isometric log-ratio coordinates) and momentary affective states

Outcome	Type	Isometric log-ratio predictor	Interpretation	Unadjusted	Adjusted
				**Coefficient (95% confidence intervals)**	**Coefficient (95% confidence intervals)**
**Happiness**	*Between-person level*	${\text{ilr}}_{1,{\text{SB}}}$	Longer SB, relative to LPA, MVPA and SLEEP	−0.39 (−1.73, 0.91)	−1.54 (−3.05, −0.05)
		${\text{ilr}}_{1,LPA}$	Longer LPA, relative to SB, MVPA and SLEEP	0.37 (−0.33, 1.05)	0.48 (−0.28, 1.23)
		${\text{ilr}}_{1,MVPA}$	Longer MVPA, relative to SB, LPA and SLEEP	0.1 (−0.07, 0.28)	0.15 (−0.04, 0.34)
		${\text{ilr}}_{1,Sleep}$	Longer SLEEP, relative to SB, LPA and MVPA	−0.08 (−1.5, 1.31)	0.90 (−0.68, 2.46)
	*Within-person level*	${\text{ilr}}_{1,{\text{SB}}}$	Longer than usual SB, relative to LPA, MVPA and SLEEP	0.09 (−0.53, 0.69)	0.09 (−0.5, 0.66)
		${\text{ilr}}_{1,LPA}$	Longer than usual LPA, relative to SB, MVPA and SLEEP	0.23 (−0.27, 0.72)	0.23 (−0.27, 0.71)
		${\text{ilr}}_{1,MVPA}$	Longer than usual MVPA, relative to SB, LPA and SLEEP	0.05 (−0.03, 0.13)	0.06 (−0.03, 0.13)
		${\text{ilr}}_{1,Sleep}$	Longer than usual SLEEP, relative to SB, LPA and MVPA	−0.35 (−1.11, 0.36)	−0.37 (−1.09, 0.31)
**Anxiousness**	*Between-person level*	${\text{ilr}}_{1,{\text{SB}}}$	Longer SB, relative to LPA, MVPA and SLEEP	1.03 (−0.55, 2.72)	1.89 (0.01, 3.78)
		${\text{ilr}}_{1,LPA}$	Longer LPA, relative to SB, MVPA and SLEEP	−0.07 (−0.91, 0.76)	−0.04 (−1.01, 0.89)
		${\text{ilr}}_{1,MVPA}$	Longer MVPA, relative to SB, LPA and SLEEP	−0.27 (−0.5, −0.05)	−0.3 (−0.55, −0.05)
		${\text{ilr}}_{1,Sleep}$	Longer SLEEP, relative to SB, LPA and MVPA	−0.79 (−2.55, 1.02)	−1.55 (−3.56, 0.41)
	*Within-person level*	${\text{ilr}}_{1,{\text{SB}}}$	Longer than usual SB, relative to LPA, MVPA and SLEEP	0.5 (−0.08, 1.09)	0.5 (−0.09, 1.07)
		${\text{ilr}}_{1,LPA}$	Longer than usual LPA, relative to SB, MVPA and SLEEP	0.16 (−0.3, 0.61)	0.15 (−0.32, 0.62)
		${\text{ilr}}_{1,MVPA}$	Longer than usual MVPA, relative to SB, LPA and SLEEP	−0.05 (−0.12, 0.03)	−0.05 (−0.12, 0.03)
		${\text{ilr}}_{1,Sleep}$	Longer than usual SLEEP, relative to SB, LPA and MVPA	−0.6 (−1.32, 0.1)	−0.6 (−1.3, 0.11)
**Tiredness**	*Between-person level*	${\text{ilr}}_{1,{\text{SB}}}$	Longer SB, relative to LPA, MVPA and SLEEP	−1.32 (−2.9, 0.19)	−0.88 (−2.58, 0.93)
		${\text{ilr}}_{1,LPA}$	Longer LPA, relative to SB, MVPA and SLEEP	0.53 (−0.3, 1.36)	0.54 (−0.3, 1.42)
		${\text{ilr}}_{1,MVPA}$	Longer MVPA, relative to SB, LPA and SLEEP	−0.23 (−0.44, −0.02)	−0.24 (−0.46, −0.02)
		${\text{ilr}}_{1,Sleep}$	Longer SLEEP, relative to SB, LPA and MVPA	0.97 (−0.72, 2.59)	0.54 (−1.32, 2.41)
	*Within-person level*	${\text{ilr}}_{1,{\text{SB}}}$	Longer than usual SB, relative to LPA, MVPA and SLEEP	0.05 (−0.73, 0.85)	0.07 (−0.71, 0.86)
		${\text{ilr}}_{1,LPA}$	Longer than usual LPA, relative to SB, MVPA and SLEEP	−0.28 (−0.94, 0.35)	−0.3 (−0.93, 0.35)
		${\text{ilr}}_{1,MVPA}$	Longer than usual MVPA, relative to SB, LPA and SLEEP	−0.02 (−0.12, 0.09)	−0.02 (−0.12, 0.09)
		${\text{ilr}}_{1,Sleep}$	Longer than usual SLEEP, relative to SB, LPA and MVPA	0.23 (−0.72, 1.2)	0.26 (−0.65, 1.18)

ilr_1_ = isometric log-ratio, is the first isometric log-ratio coordinate representing each time-use behaviour relative to the remaining behaviours; *SB* Sedentary behaviour, *LPA* Light-intensity physical activity, *MVPA* Moderate-to-vigorous intensity physical activity

Models were adjusted for age range, gender, ethnicity and NZ deprivation index

**Table 4:** Relationship between time-use compositions (expressed as isometric log-ratio coordinates) and life satisfaction

			**Unadjusted**			**Adjusted**		
**Outcome**	**Isometric log-ratio predictor**	**Interpretation**	**Coefficient**	**p value**	**R squared**	**Coefficient**	**p value**	**R squared**
**Life satisfaction**	${\text{ilr}}_{1,{\text{SB}}}$	Longer SB, relative to LPA, MVPA and SLEEP	−0.08	0.85	−0.005	−0.30	0.53	0.03
	${\text{ilr}}_{1,LPA}$	Longer LPA, relative to SB, MVPA and SLEEP	0.02	0.93		0.12	0.66	
	${\text{ilr}}_{1,MVPA}$	Longer MVPA, relative to SB, LPA and SLEEP	0.07	0.25		0.11	0.10	
	${\text{ilr}}_{1,Sleep}$	Longer SLEEP, relative to SB, LPA and MVPA	−0.01	0.98		0.07	0.90	

ilr_1_ = isometric log-ratio, is the first isometric log-ratio coordinate representing each time-use behaviour relative to the remaining behaviours; *SB* Sedentary behaviour, *LPA* Light-intensity physical activity, *MVPA* Moderate-to-vigorous intensity physical activity

Models were adjusted for age range, gender, ethnicity and NZ deprivation index

**Fig. 1:**
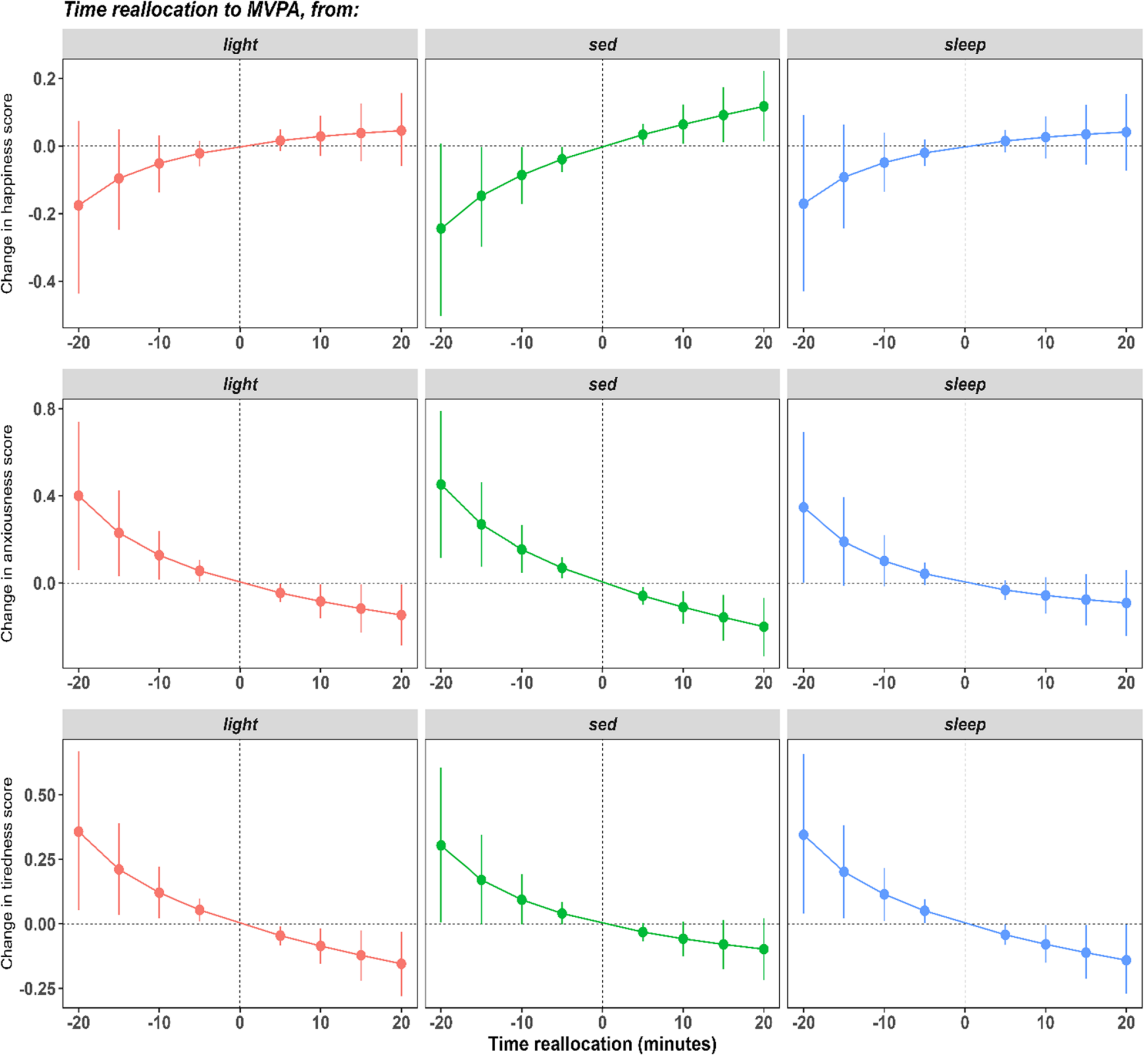
Estimated changes in happiness, anxiousness and tiredness scores associated with reallocating ± 20 min, in 5-min increments, to or from MVPA at the between-person level. Note: Negative minutes indicate time reallocated from MVPA to the other behaviour; positive minutes indicate time reallocated to MVPA from the other behaviour. sed – sedentary behaviour; light – Light physical Activity; MVPA – Moderate-to-vigorous physical activity

Finally, Tables 5, 6 and 7 present the results of the substitution analysis, which explored the predicted differences in affect at the between-person level. The results indicate that more LPA or MVPA at the expense of sedentary behaviour was associated with higher happiness, a trend observed across all reallocations ranging from 5 to 20 min, with the greatest difference noted at 20 min (0.12, CI = [0.01, 0.22]). Conversely, more sedentary behaviour at the expense of MVPA or LPA was associated with lower happiness. Additionally, more sedentary behaviour and less MVPA was associated with increased anxiousness, with associations across all reallocations. Figure 1 illustrates the predicted differences in happiness, anxiousness, and tiredness scores associated with reallocating time spent in sedentary behaviour, LPA, and sleep to or from MVPA at the between-person level. The substitution analysis for tiredness revealed different patterns; less MVPA compensated by LPA or sleep was associated with higher tiredness scores across all reallocations, while more MVPA at the expense of LPA or sleep was associated with decreased tiredness scores. No statistically significant reallocations were found at the within-person level or in the substitution models exploring life satisfaction. These results are presented in supplementary Tables 8, 9, 10 and 11 respectively.

**Table 5:** Predicted change (95% CI) in happiness following reallocation of time between various physical behaviours (between-person level)

		***Allocated to…***																
		*SB*					*LPA*				*MVPA*				*Sleep*			
		***5*** ***mins***	***10*** ***mins***		***15 min***	***20 min***	***5*** ***mins***	***10 min***	***15 min***	***20 min***	***5*** ***mins***	***10 min***	***15 min***	***20 min***	***5*** ***mins***	***10 min***	***15 min***	***20 min***
***Taken from…***	*SB*	*Not Applicable*					0.02 (0.00, 0.03)	0.03 (0.00, 0.07)	0.05 (0.00, 0.10)	0.07 (0.01, 0.13)	0.03 (0.00, 0.07)	0.06 (0.01, 0.12)	0.09 (0.01, 0.17)	0.12 (0.01, 0.22)	0.02 (0.00, 0.04)	0.04 (0.00, 0.09)	0.06 (0.00, 0.13)	0.08 (0.00, 0.17)
	*LPA*	−0.02 (−0.03, 0.00)	−0.03 (−0.07, −0.00)	−0.05 (−0.1, −0.00)		**-**0.07 (−0.13, −0.01)	*Not Applicable*				0.02 (−0.02, 0.05)	0.03 (−0.03, 0.09)	0.04 (−0.05, 0.13)	0.05 (−0.06, 0.16)	0.00 (−0.02, 0.02)	0.00 (−0.04, 0.04)	0.00 (−0.06, 0.07)	0.00 (−0.08, 0.09)
	*MVPA*	−0.04 (−0.08, 0.00)	−0.09 (−0.17, −0.00)	−0.15 (−0.3, −0.00)		−0.24 (−0.50, 0.00)	−0.02 (−0.06, 0.01)	−0.05 (−0.14, 0.03)	−0.10 (−0.25, 0.05)	−0.18 (−0.44, 0.08)	*Not Applicable*				−0.02 (−0.06, 0.02)	−0.05 (−0.14, 0.04)	−0.09 (−0.24, 0.06)	−0.17 (−0.43, 0.09)
	*Sleep*	−0.02 (−0.04, 0.00)	−0.04 (−0.08, 0.01)	−0.06 (−0.13, 0.01)		−0.07 (−0.17, 0.02)	0.00 (−0.02, 0.02)	0.00 (−0.04, 0.04)	0.00 (−0.07, 0.06)	−0.01 (−0.09, 0.07)	0.02 (−0.02, 0.05)	0.03 (−0.04, 0.09)	0.04 (−0.05, 0.12)	0.04 (−0.07, 0.15)	*Not Applicable*			

*SB* Sedentary behaviour, *LPA* Light-intensity physical activity, *MVPA* Moderate-to-vigorous intensity physical activity

Models were adjusted for age range, gender, ethnicity and NZ Deprivation index. Bold values represent significant associations

**Table 6:** Predicted change (95% CI) in anxiousness following reallocation of time between various physical behaviours (between-person level)

		***Allocated to…***																
		*SB*					*LPA*				*MVPA*				*Sleep*			
		***5*** ***mins***	***10*** ***mins***		***15 min***	***20 min***	***5*** ***mins***	***10 min***	***15 min***	***20 min***	***5*** ***mins***	***10 min***	***15 min***	***20 min***	***5*** ***mins***	***10 min***	***15 min***	***20 min***
***Taken from…***	*SB*	*Not Applicable*					−0.01 (−0.03, 0.01)	−0.03 (−0.07, 0.01)	−0.04 (−0.10, 0.02)	−0.05 (−0.14, 0.03)	−0.06 (−0.10, −0.02)	−0.11 (−0.19, −0.04)	−0.16 (−0.27, −0.05)	−0.20 (−0.34, −0.07)	−0.03 (−0.06, 0.00)	−0.05 (−0.11, 0.01)	−0.08 (−0.17, 0.01)	−0.11 (−0.23, 0.01)
	*LPA*	0.01 (−0.01, 0.03)	0.03 (−0.02, 0.07)	0.04 (−0.02, 0.10)		0.05 (−0.03, 0.14)	*Not Applicable*				−0.05 (−0.09, −0.00)	−0.08 (−0.16, −0.01)	−0.12 (−0.23, −0.01)	−0.15 (−0.29, −0.01)	−0.01 (−0.04, 0.01)	−0.03 (−0.08, 0.03)	−0.04 (−0.12, 0.04)	−0.05 (−0.16, 0.05)
	*MVPA*	0.07 (0.02, 0.12)	0.15 (0.05, 0.26)	0.27 (0.08, 0.46)		0.45 (0.12, 0.79)	0.06 (0.01, 0.11)	0.13 (0.02, 0.24)	0.23 (0.03, 0.43)	0.40 (0.06, 0.74)	*Not Applicable*				0.04 (−0.01, 0.10)	0.10 (−0.02, 0.22)	0.19 (−0.01, 0.39)	0.35 (0.00, 0.69)
	*Sleep*	0.03 (−0.00, 0.06)	0.05 (−0.01, 0.11)	0.08 (−0.01, 0.17)		0.11 (−0.02, 0.23)	0.01 (−0.01, 0.04)	0.03 (−0.03, 0.08)	0.04 (−0.04, 0.12)	0.06 (−0.05, 0.16)	−0.03 (−0.08, 0.01)	−0.06 (−0.14, 0.03)	−0.08 (−0.20, 0.04)	−0.09 (−0.24, 0.06)	*Not Applicable*			

*SB* Sedentary behaviour, *LPA* Light-intensity physical activity, *MVPA* Moderate-to-vigorous intensity physical activity

Models were adjusted for age range, gender, ethnicity and NZ Deprivation index. Bold values represent significant associations

**Table 7:** Predicted change (95% CI) in tiredness following reallocation of time between various physical behaviours (between-person level)

		***Allocated to…***																
		*SB*					*LPA*				*MVPA*				*Sleep*			
		***5*** ***mins***	***10*** ***mins***		***15 min***	***20 min***	***5*** ***mins***	***10 min***	***15 min***	***20 min***	***5*** ***mins***	***10 min***	***15 min***	***20 min***	***5*** ***mins***	***10 min***	***15 min***	***20 min***
***Taken from…***	*SB*	*Not Applicable*					0.01 (−0.01, 0.03)	0.03 (−0.01, 0.06)	0.04 (−0.01, 0.10)	0.05 (−0.02, 0.13)	−0.03 (−0.07, 0.00)	−0.06 (−0.13, 0.01)	−0.08 (−0.17, 0.02)	−0.10 (−0.22, 0.02)	0.01 (−0.02, 0.04)	0.02 (−0.03, 0.08)	0.03 (−0.05, 0.12)	0.04 (−0.07, 0.16)
	*LPA*	−0.01 (−0.03, 0.00)	−0.03 (−0.06, 0.01)	−0.04 (−0.10, 0.01)		−0.06 (−0.13, 0.02)	*Not Applicable*				−0.05 (−0.08, −0.01)	−0.09 (−0.16, −0.02)	−0.12 (−0.22, −0.03)	−0.16 (−0.28, −0.03)	−0.00 (−0.03, 0.02)	−0.01 (−0.06, 0.04)	−0.01 (−0.08, 0.06)	−0.02 (−0.11, 0.08)
	*MVPA*	0.04 (−0.00, 0.08)	0.09 (−0.00, 0.19)	0.17 (−0.00, 0.35)		0.30 (0.01, 0.61)	0.05 (0.01, 0.10)	0.12 (0.02, 0.22)	0.21 (0.03, 0.39)	0.36 (0.05, 0.67)	*Not Applicable*				0.05 (0.00, 0.10)	0.11 (0.01, 0.22)	0.20 (0.02, 0.38)	0.35 (0.04, 0.66)
	*Sleep*	−0.01 (−0.04, 0.02)	−0.02 (−0.08, 0.03)	−0.03 (−0.12, 0.05)		−0.04 (−0.16, 0.07)	0.00 (−0.02, 0.03)	0.01 (−0.04, 0.06)	0.01 (−0.06, 0.08)	0.01 (−0.08, 0.11)	−0.04 (−0.08, 0.00)	−0.08 (−0.15, 0.00)	−0.11 (−0.21, 0.00)	−0.14 (−0.27, 0.00)	*Not Applicable*			

*SB* Sedentary behaviour, *LPA* Light-intensity physical activity, *MVPA* Moderate-to-vigorous intensity physical activity

Models were adjusted for age range, gender, ethnicity and NZ Deprivation index. Bold values represent significant associations

## Discussion

This study examined how daily time-use behaviours – sedentary behaviour, LPA, MVPA, and sleep – were associated with evaluative wellbeing (life satisfaction), and experienced wellbeing (happiness, anxiousness and tiredness). By integrating accelerometer data with real-time momentary affect assessments, the findings enhance our understanding of the relationship between time use and subjective wellbeing within the time-use epidemiology field.

### Time use and happiness

The analysis revealed a negative association between sedentary behaviour relative to other behaviours and happiness at the between-person level *(B* = − 1.54, 95% CI = −3.05 to −0.05). Specifically, individuals who engaged in higher levels of sedentary behaviour reported lower happiness scores. This finding is consistent with existing literature that emphasises the detrimental effects of prolonged sedentary activities, particularly those involving screens, on mental health [35]. Furthermore, less sedentary behaviour and higher LPA or MVPA was positively associated with happiness, with a significant increase observed as reallocation time increased from 5 to 20 min. If not attributable to reverse causation (i.e., happier individuals being more likely to engage in less sedentary behaviour and more LPA, MVPA and Sleep), this underscores the importance of even modest shifts in physical activity to enhance experienced wellbeing, supporting previous findings that suggest small, incremental increases in physical activity can yield substantial benefits [36]. Our results align well with prior research [37] demonstrating the mental health benefits of physical activity, although most studies focus on structured exercise rather than incidental or light activities [38]. Notably, the within-person analysis did not reveal daily associations between time-use behaviours and happiness. This absence could be due to the nature of the data and the way the indicators were calculated, which primarily capture average daily behaviour rather than momentary changes. Momentary affect reflects how individuals feel at the specific time of assessment, and thus, any within-person associations are likely to be tied to the immediate activity undertaken at the time of the response, rather than to daily average activity levels. This distinction suggests that the immediate impact of activities such as physical movement may be more accurately reflected by within-person analyses focusing on real-time behaviour, rather than on broader daily averages. Moreover, averaging measures across the day potentially dilutes short-term variations in both affect and behaviours, making it harder to detect associations that occur within narrower time frames. For example, brief bouts of physical activity—such as taking a walk or engaging in light stretching—may have immediate, localised effects on experienced wellbeing, but these effects are likely to be obscured when data are aggregated over the course of a day. This also highlights the importance for future studies to track momentary wellbeing and time-use behaviours in real-time. By analysing the immediate impact of specific behaviours on wellbeing, we could gain a deeper understanding of the temporal dynamics of wellbeing, bridging the gap between within-person and between-person findings.

### Time use and anxiousness

At the between-person level, increases in sedentary behaviour were significantly associated with heightened anxiousness (*B* = 1.89, 95% CI = 0.01 to 3.78). This aligns with existing literature that links sedentary behaviour to increased risks of anxiety disorders, underscoring the mental health implications of prolonged inactivity [39, 40]. Sedentary lifestyles, particularly those involving excessive screen time, can lead to decreased social interaction and increased feelings of isolation, which may worsen anxiety symptoms [41].

The substitution analysis revealed that spending 5–20 min more in LPA to MVPA at the expense of sedentary behaviour was associated with lower anxiousness. Under the assumption that reverse causality can be ruled out, this suggests that even small increases in physical activity can have a meaningful impact on anxiety levels, supporting the potential for public health interventions that encourage individuals to integrate more physical activity into their daily routines as a strategy for managing anxiety. Furthermore, emerging evidence consistently highlights the role of physical activity in enhancing resilience against stress and anxiety [42, 43]. Notably, breaking sedentary behaviour with short bouts of physical activity, regardless of intensity, may also provide similar benefits for anxiety reduction [44]. Future studies could investigate which specific types of physical activities are most effective in reducing anxiety, considering factors such as exercise duration, intensity, and social context.

### Time-use and tiredness

Our results demonstrated a significant negative association between MVPA and tiredness (*B* = −0.24, 95% CI = −0.46 to −0.02), suggesting that engaging in higher intensity physical activity might alleviate feelings of fatigue. This finding aligns with existing research that indicates physical activity can enhance energy levels and combat feelings of tiredness by improving overall physical fitness and cardiovascular health [45]. Although a clear biological explanation for the link between physical activity and reduced fatigue has not been fully established, studies have hypothesised that regular participation in physical activities alter neurotransmitter and neuromodulator levels, thereby contributing to reduced fatigue [46, 47]. Additionally, our analysis indicated that more MVPA at the expense of LPA or sleep was associated with reduced tiredness, with this effect becoming more pronounced as the duration of reallocation increased from 5 to 20 min. This trend emphasises the importance of moderate-to-vigorous activity not only in managing tiredness but also in enhancing energy and vitality throughout the day. Engaging in even short bouts of MVPA may serve as an effective intervention for individuals experiencing fatigue, especially in the context of sedentary lifestyles.

Inadequate sleep has been linked to increased feelings of tiredness and lower levels of physical activity, creating a vicious cycle that can negatively impact overall wellbeing [48]. However, evidence regarding this relationship is mixed. A study by Harris et al. (2020) suggests that sleep quality is a significant predictor of daily fatigue ratings, rather than total sleep time [49]. Our findings indicate that reallocating time from MVPA to sleep is associated with greater tiredness. This relationship remains unclear; it may be that the average sleep duration in our sample was already around 8 h, which falls within the optimal range for adults [50], so additional sleep might not necessarily reduce tiredness. Alternatively, the average duration of MVPA in our sample was only 8 min which is likely to be well below the optimal level suggesting a high return on reallocating any time to MVPA. Another potential explanation is the directionality of these associations, which was not examined in this study. It is possible that higher tiredness leads to more sleep, rather than sleep reducing tiredness. Future studies should consider the temporal order of these behaviours, such as whether sleep the previous night predicts tiredness the following day or whether time-use patterns over the 24 h prior to an EMA survey predict how it is answered, to clarify these relationships. Additionally, our analysis did not assess sleep quality, which could be an important factor influencing fatigue. Future research should explore the interplay between sleep quality, physical activity, and tiredness to better understand how these factors interact and contribute to overall wellbeing.

### Life satisfaction

Contrary to our expectations, no associations were found between time-use behaviours and life satisfaction. The small sample size and low variability in life satisfaction within our sample, with approximately 60% of participants rating their satisfaction between 7 and 9, may have limited our ability to detect significant associations with time-use behaviours. While some studies argue that high levels of sedentary behaviour may diminish life satisfaction due to associated health risks, others report weak or non-existent relationships with subjective wellbeing [51].

This finding contrasts with the well-documented link between physical activity and overall life satisfaction in the literature [52]. For instance, a 2014 study found that individuals engaging in frequent MVPA during a week reported higher overall satisfaction with life, although these were self-reported measures [53]. It is important to note that the majority of studies exploring these relationships rely on self-reported measures of MVPA rather than objective measures [16, 54, 55], which can be subject to bias and overestimation [56]. While accelerometery provides more objective data, it comes with its own set of assumptions, which can result in different estimates and relationships. Self-report, on the other hand, captures the lived experience of time use, reflecting how individuals perceive and engage in activities. Combining both methods can offer a fuller understanding of wellbeing, capturing both subjective experiences and objective measures of physical activity.

One other possible explanation for the discrepancy in our results is the methodological approach used in this study. Many previous studies did not account for the compositional nature of time-use data, where the distribution of time across different behaviours collectively affects the outcome. For instance, while one study demonstrated a significant association between quality of life and MVPA, it examined MVPA in isolation rather than considering the composition of other behaviours [57]. By considering the interactions among different behaviours, our analysis may uncover subtle effects that are often overlooked in non-compositional studies, as evidenced by a comparative study that found significant differences in results when using compositional data analysis versus standard methods to examine age and gender differences in sedentary time and physical activity at work [58]. Additionally, the context, and domain of physical activity might influence its impact on life satisfaction [55]. Our study captured only the total amount of physical activity without distinguishing between different domains such as leisure-time, or occupational. Physical activity performed during leisure time, which is often voluntary and enjoyable, may have a stronger positive effect on life satisfaction compared to physical activity that is obligatory or work-related [55]. Therefore, simply measuring the amount of physical activity may not be sufficient to elucidate the relationship between MVPA and life satisfaction. Lastly, life satisfaction, being a more stable and global measure of wellbeing, may be influenced by factors beyond daily activity patterns, such as social relationships, economic stability, and long-term health outcomes, and physical activity may have only an extra marginal contribution to overall life satisfaction.

### Limitations and future research

Despite the strengths of our study, including the use of objective accelerometery data and real-time wellbeing assessments, several limitations should be acknowledged. The most important of these is the relatively small sample size—particularly with respect to measures of experienced wellbeing linked to MVPA. Furthermore, the sample was not fully representative of the New Zealand adult population, with an overrepresentation of women and certain ethnic groups. While we adjusted for gender, age, and ethnicity in our models, this limits the generalisability of the findings to the wider population. Next, the relatively short duration of accelerometer wear time (mean of 4.4 days) may not fully capture participants'habitual behaviour patterns, potentially limiting the generalisability of our findings. Our analysis did not explore how environmental context—such as social interactions and environmental factors—might mediate the relationship between time-use behaviours and wellbeing. Further research could include a more detailed examination of different types of physical behaviours (such as cycling, running, or standing) as these measures provide additional context, beyond activity intensity. Additionally, distinguishing the context of sedentary time—for example, differentiating between activities like reading versus screen time might offer new insights. Another limitation is that, due to our time-use and physical activity indicators that were aggregated either at the week level or day level, the directionality of these associations could not be examined, meaning that causality cannot be optimally inferred from our findings. Future research should explore the temporal relationships between physical activity, sedentary behaviour, and wellbeing to better understand the causal pathways. Lastly, our EMA prompts were restricted to the period between 8:00 am and 9:00 pm to avoid disturbing participants during typical sleep hours. While this ensured consistency and reduced participant burden, it may have excluded key emotional periods such as the first hour after waking and the hour before sleep—times often marked by distinct affective states. Future studies may benefit from capturing these early and late time windows to better understand the full daily rhythm of experienced wellbeing. Furthermore, although EMA prompts were randomly distributed across all days of the week, we did not specifically examine differences between weekdays and weekends. Given that affective experiences may vary by day, future research could explore these temporal patterns in more detail.

## Conclusion

Our study explored the association between daily time-use behaviours and different measures of subjective wellbeing. We found that reducing sedentary behaviour and increasing physical activity, even at light intensity, was positively associated with happiness, though these effects only emerged at the between-person level. Additionally, more MVPA was associated with decreased anxiousness and tiredness, highlighting the interconnectedness of these experiences. Although some significant associations were observed, most tested relationships were either modest in magnitude or not statistically significant. This suggests that factors beyond physical behaviours—such as the social, environmental, and contextual aspects of time use—may play a more influential role in shaping wellbeing. This highlights the need for future research to examine how broader factors like social relationships and environmental context contribute to daily experiences of wellbeing. Public health interventions aimed at enhancing wellbeing should prioritise reducing sedentary time and promoting physical activity, including those with light intensity. The next step is to explore sequential associations, such as time-use behaviours occurring immediately or in the 24 h before a momentary wellbeing response is recorded, as this may reveal how these immediate activities influence momentary happiness, anxiousness, and tiredness.

## Supplementary Information

Supplementary Material 1.

## Data Availability

The datasets generated and analysed during the current study are not publicly available due to ethical reasons, but are available from the corresponding author on reasonable request.

## Abbreviations

SB: Sedentary behaviour
LPA: Light physical activity
MVPA: Moderate-vigorous physical activity
EMA: Ecological momentary assessment
NZDep2018: 2018 New Zealand Deprivation index
Ilr: Isometric log-ratio
SBP: Sequential binary partition
SD: Standard deviation
GSD: Geometric standard deviation
CI: Confidence interval
