# Correction: Daily time-use compositions of physical behaviours and its association with evaluative and experienced wellbeing: a multilevel compositional analysis

**DOI:** 10.1186/s12966-025-01790-z

**Published:** 2025-07-18

**Authors:** Anantha Narayanan, Scott Duncan, Conal Smith, Flora Le, Lisa Mackay, Julia McPhee, Basile Chaix, Tom Stewart

**Affiliations:** 1 School of Sport and Recreation, Auckland University of Technology, Private Bag 92006, Auckland, 1142 New Zealand; 2 Kōtātā Insight, Wellington, New Zealand; 3 School of Psychological Sciences, Monash University, Melbourne, Australia; 4 Institut Pierre Louis d’Épidémiologie Et de Santé Publique, Nemesis Research Team, Sorbonne University, Paris, France

**Narayanan et al. Int J Behav Nutr Phys Act (2025) 22:73**

10.1186/s12966-025-01769-w

Following the publication of the original article, the authors reported that the names of two authors were misspelled:

- “Conal Smlith” should be “Conal Smith”.
- “Basile Chax” should be “Basile Chaix”.

The authorship list as shown in this article is correct. The original article has been updated accordingly.

