# Supplementary material for: Daily time-use compositions of physical behaviours and its association with evaluative and experienced wellbeing: a multilevel compositional analysis
Source: Int J Behav Nutr Phys Act. 2025 Jun 11;22:73. doi: 10.1186/s12966-025-01769-w (PMC12160111; doi:10.1186/s12966-025-01769-w)
Supplement: Supplementary file 1 — Supplementary Material 1. [file 12966_2025_1769_MOESM1_ESM.docx]

## Supplementary tables

**Table 8.** Predicted change (95% CI) in happiness following reallocation of time between various physical behaviours (within-person level).

|  | | ***Allocated to…*** | | | | | | | | | | | | | | | |
| --- | --- | --- | --- | --- | --- | --- | --- | --- | --- | --- | --- | --- | --- | --- | --- | --- | --- |
|  |  | *SB* | | | | *LPA* | | | | *MVPA* | | | | *Sleep* | | | |
|  |  | ***5  mins*** | ***10 mins*** | ***15 mins*** | ***20 mins*** | ***5  mins*** | ***10 mins*** | ***15 mins*** | ***20 mins*** | ***5  mins*** | ***10 mins*** | ***15 mins*** | ***20 mins*** | ***5  mins*** | ***10 mins*** | ***15 mins*** | ***20 mins*** |
| ***Taken from…*** | *SB* | *Not Applicable* | | | | 0.003 ( -0.006, 0.012) | 0.005 ( -0.013, 0.023) | 0.008 ( -0.019, 0.034) | 0.011 ( -0.025, 0.046) | 0.008 (-0.005, 0.021) | 0.014 (-0.009, 0.038) | 0.02 (-0.013, 0.053) | 0.025 (-0.016, 0.067) | -0.004 ( -0.014, 0.006) | -0.008 (-0.027, 0.011) | -0.012 (-0.04, 0.017) | -0.016 (-0.054, 0.023) |
|  | *LPA* | -0.003 ( -0.012, 0.006) | -0.006 (-0.024, 0.013) | -0.009 (-0.036, 0.019) | -0.012 (-0.048, 0.026) | *Not Applicable* | | | | 0.005 (-0.01, 0.021) | 0.009 (-0.02, 0.038) | 0.011 (-0.03, 0.053) | 0.013 (-0.04, 0.067) | -0.007 (-0.019, 0.005) | -0.014 (-0.038, 0.01) | -0.021 (-0.057, 0.015) | -0.028 (-0.076, 0.019) |
|  | *MVPA* | -0.01 (-0.025, 0.005) | -0.023 (-0.057, 0.011) | -0.041 (-0.103, 0.02) | -0.072 (-0.18, 0.034) | -0.007 (-0.025, 0.01) | -0.017 (-0.057, 0.022) | -0.033 (-0.103, 0.035) | -0.061 (-0.18, 0.053) | *Not Applicable* | | | | -0.014 (-0.031, 0.002) | -0.031 (-0.068, 0.005) | -0.053 (-0.118, 0.011) | -0.088 (-0.2, 0.021) |
|  | *Sleep* | 0.004 (-0.006, 0.014) | 0.008 (-0.011, 0.027) | 0.012 (-0.017, 0.041) | 0.017 (-0.023, 0.055) | 0.007 (-0.005, 0.019) | 0.014 (-0.01, 0.037) | 0.021 (-0.015, 0.056) | 0.027 (-0.019, 0.074) | 0.012 (-0.002, 0.026) | 0.023 (-0.003, 0.05) | 0.033 (-0.004, 0.072) | 0.042 (-0.005, 0.091) | *Not Applicable* | | | |

SB = Sedentary behaviour; LPA= Light-intensity physical activity; MVPA= Moderate-to-vigorous intensity physical activity. Models were adjusted for age range, gender, ethnicity and NZ Deprivation index.

|  | | ***Allocated to…*** | | | | | | | | | | | | | | | | |
| --- | --- | --- | --- | --- | --- | --- | --- | --- | --- | --- | --- | --- | --- | --- | --- | --- | --- | --- |
|  |  | *SB* | | | | | *LPA* | | | | *MVPA* | | | | *Sleep* | | | |
|  |  | ***5  mins*** | ***10***  ***mins*** | | ***15 mins*** | ***20 mins*** | ***5  mins*** | ***10 mins*** | ***15 mins*** | ***20 mins*** | ***5  mins*** | ***10 mins*** | ***15 mins*** | ***20 mins*** | ***5  mins*** | ***10 mins*** | ***15 mins*** | ***20 mins*** |
| ***Taken from…*** | *SB* | *Not Applicable* | | | | | -0.001 (-0.01, 0.008) | -0.002 (-0.019, 0.015) | -0.003 (-0.028, 0.022) | -0.004 (-0.038, 0.029) | -0.011 (-0.023, 0.002) | -0.02 (-0.043, 0.004) | -0.029 (-0.061, 0.005) | -0.037 (-0.078, 0.005) | -0.009 (-0.018, 0.001) | -0.018 (-0.036, 0.002) | -0.026 (-0.054, 0.002) | -0.035 (-0.072, 0.003) |
|  | *LPA* | 0.001 (-0.008, 0.01) | 0.002 (-0.016, 0.019) | 0.003 (-0.024, 0.029) | | 0.003 (-0.032, 0.038) | *Not Applicable* | | | | -0.01 (-0.025, 0.006) | -0.018 (-0.047, 0.011) | -0.026 (-0.068, 0.016) | -0.034 (-0.087, 0.022) | -0.008 (-0.02, 0.003) | -0.016 (-0.04, 0.007) | -0.023 (-0.06, 0.011) | -0.031 (-0.08, 0.014) |
|  | *MVPA* | 0.012 (-0.003, 0.027) | 0.027 (-0.008, 0.061) | 0.047 (-0.016, 0.108) | | 0.077 (-0.031, 0.182) | 0.011 (-0.007, 0.029) | 0.025 (-0.015, 0.064) | 0.044 (-0.027, 0.111) | 0.073 (-0.046, 0.189) | *Not Applicable* | | | | 0.004 (-0.013, 0.02) | 0.01 (-0.027, 0.047) | 0.021 (-0.045, 0.086) | 0.043 (-0.07, 0.155) |
|  | *Sleep* | 0.009 (-0.001, 0.018) | 0.018 (-0.001, 0.036) | 0.026 (-0.002, 0.055) | | 0.035 (-0.003, 0.073) | 0.008 (-0.003, 0.02) | 0.016 (-0.007, 0.04) | 0.024 (-0.01, 0.06) | 0.031 (-0.013, 0.079) | -0.002 (-0.016, 0.012) | -0.003 (-0.028, 0.024) | -0.002 (-0.039, 0.035) | -0.002 (-0.049, 0.047) | *Not Applicable* | | | |

**Table 9.** Predicted change (95% CI) in anxiousness following reallocation of time between various physical behaviours (within-person level).

**Table 10.** Predicted change (95% CI) in tiredness following reallocation of time between various physical behaviours (within-person level).

|  | | ***Allocated to…*** | | | | | | | | | | | | | | | | |
| --- | --- | --- | --- | --- | --- | --- | --- | --- | --- | --- | --- | --- | --- | --- | --- | --- | --- | --- |
|  |  | *SB* | | | | | *LPA* | | | | *MVPA* | | | | *Sleep* | | | |
|  |  | ***5  mins*** | ***10***  ***mins*** | | ***15 mins*** | ***20 mins*** | ***5  mins*** | ***10 mins*** | ***15 mins*** | ***20 mins*** | ***5  mins*** | ***10 mins*** | ***15 mins*** | ***20 mins*** | ***5  mins*** | ***10 mins*** | ***15 mins*** | ***20 mins*** |
| ***Taken from…*** | *SB* | *Not Applicable* | | | | | -0.01 (-0.033, 0.014) | -0.014 (-0.049, 0.021) | -0.019 (-0.065, 0.028) | -0.01 (-0.033, 0.014) | -0.007 (-0.037, 0.024) | -0.009 (-0.053, 0.034) | -0.012 (-0.066, 0.043) | -0.007 (-0.037, 0.024) | 0.004 (-0.021, 0.029) | 0.006 (-0.031, 0.043) | 0.008 (-0.041, 0.058) | 0.004 (-0.021, 0.029) |
|  | *LPA* | 0.01 (-0.014, 0.034) | 0.015 (-0.022, 0.051) | 0.02 (-0.029, 0.069) | | 0.01 (-0.014, 0.034) | *Not Applicable* | | | | 0.003 (-0.035, 0.042) | 0.006 (-0.05, 0.061) | 0.009 (-0.063, 0.08) | 0.003 (-0.035, 0.042) | 0.014 (-0.018, 0.046) | 0.021 (-0.027, 0.07) | 0.028 (-0.037, 0.093) | 0.014 (-0.018, 0.046) |
|  | *MVPA* | 0.009 (-0.035, 0.055) | 0.017 (-0.062, 0.097) | 0.028 (-0.11, 0.168) | | 0.009 (-0.035, 0.055) | 0 (-0.052, 0.051) | 0.002 (-0.089, 0.092) | 0.01 (-0.142, 0.159) | 0 (-0.052, 0.051) | *Not Applicable* | | | | 0.013 (-0.036, 0.061) | 0.022 (-0.064, 0.105) | 0.036 (-0.112, 0.179) | 0.013 (-0.036, 0.061) |
|  | *Sleep* | -0.004 (-0.029, 0.021) | -0.006 (-0.044, 0.031) | -0.008 (-0.059, 0.042) | | -0.004 (-0.029, 0.021) | -0.014 (-0.046, 0.018) | -0.02 (-0.068, 0.027) | -0.027 (-0.091, 0.037) | -0.014 (-0.046, 0.018) | -0.01 (-0.044, 0.024) | -0.015 (-0.063, 0.034) | -0.02 (-0.081, 0.043) | -0.01 (-0.044, 0.024) | *Not Applicable* | | | |

**Table 11**. Predicted change (95% CI) in life satisfaction following reallocation of time between various physical behaviours.

|  | | ***Allocated to…*** | | | | | | | | | | | | | | | |
| --- | --- | --- | --- | --- | --- | --- | --- | --- | --- | --- | --- | --- | --- | --- | --- | --- | --- |
|  |  | *SB* | | | | *Light* | | | | *MVPA* | | | | *Sleep* | | | |
|  |  | ***5  mins*** | ***10 mins*** | ***15 mins*** | ***20 mins*** | ***5  mins*** | ***10 mins*** | ***15 mins*** | ***20 mins*** | ***5  mins*** | ***10 mins*** | ***15 mins*** | ***20 mins*** | ***5  mins*** | ***10 mins*** | ***15 mins*** | ***20 mins*** |
| ***Taken from…*** | *SB* | *Not Applicable* | | | | -0.004 (-0.015, 0.007) | -0.008 (-0.030, 0.015) | -0.012 (-0.045, 0.022) | -0.015 (-0.060, 0.029) | -0.238 (-0.518, 0.043) | 0.430 (-0.092, 0.952) | 0.428 (-0.095, 0.952) | 0.427 (-0.099, 0.952) | -0.003 (-0.018, 0.013) | -0.005 (-0.036, 0.026) | -0.008 (-0.053, 0.038) | -0.010 (-0.071, 0.051) |
|  | *LPA* | 0.004 (-0.007, 0.015) | 0.008 (-0.014, 0.029) | 0.011 (-0.021, 0.044) | 0.015 (-0.028, 0.058) | *Not Applicable* | | | | -0.234 (-0.517, 0.050) | 0.438 (-0.078, 0.955) | 0.441 (-0.075, 0.957) | 0.443 (-0.073, 0.958) | 0.001 (-0.014, 0.016) | 0.002 (-0.027, 0.032) | 0.004 (-0.041, 0.048) | 0.005 (-0.055, 0.064) |
|  | *MVPA* | 0.066 (-0.009, 0.140) | 0.106 (-0.014, 0.225) | 0.135 (-0.016, 0.287) | 0.159 (-0.017, 0.335) | 0.062 (-0.016, 0.140) | 0.098 (-0.028, 0.224) | 0.124 (-0.038, 0.285) | 0.143 (-0.047, 0.333) | *Not Applicable* | | | | 0.063 (-0.016, 0.142) | 0.101 (-0.028, 0.229) | 0.128 (-0.037, 0.293) | 0.149 (-0.046, 0.343) |
|  | *Sleep* | 0.003 (-0.013, 0.018) | 0.005 (-0.025, 0.035) | 0.008 (-0.038, 0.053) | 0.010 (-0.050, 0.071) | -0.001 (-0.016, 0.014) | -0.003 (-0.033, 0.027) | -0.004 (-0.049, 0.041) | -0.005 (-0.066, 0.055) | -0.235 (-0.520, 0.049) | 0.435 (-0.079, 0.949) | 0.436 (-0.076, 0.949) | 0.437 (-0.074, 0.948) | *Not Applicable* | | | |

SB = Sedentary behaviour; LPA= Light-intensity physical activity; MVPA= Moderate-to-vigorous intensity physical activity.
Models were adjusted for age range, gender, ethnicity and NZ Deprivation index.
